# Therapeutic interventions in severe asthma

**DOI:** 10.1186/s40413-016-0130-3

**Published:** 2016-11-28

**Authors:** Giorgio Walter Canonica, Gianenrico Senna, Patrick D. Mitchell, Paul M. O’Byrne, Giovanni Passalacqua, Gilda Varricchi

**Affiliations:** 1 Allergy & Respiratory Disease Clinic, DIMI Department of Internal Medicine, IRCCS AOU San Martino-IST, University of Genoa, Genova, Italy; 2 Allergy Unit, Verona University and General Hospital, Verona, Italy; 3 Firestone Institute of Respiratory Health and Department of Medicine, Michael G DeGroote School of Medicine, McMaster University, Hamilton, Ontario Canada; 4 Department of Translational Medical Sciences, Division of Clinical Immunology and Allergy, University of Naples Federico II, Naples, Italy

**Keywords:** Severe asthma, Phenotypes, Biological therapeutics, Eosinophils

## Abstract

The present paper addresses severe asthma which is limited to 5-10% of the overall population of asthmatics. However, it accounts for 50% or more of socials costs of the disease, as it is responsible for hospitalizations and Emergency Department accesses as well as expensive treatments.

The recent identification of different endotypes of asthma, based on the inflammatory pattern, has led to the development of tailored treatments that target different inflammatory mediators. These are major achievements in the perspective of Precision Medicine: a leading approach to the modern treatment strategy.

Omalizumab, an anti-IgE antibody, has been the only biologic treatment available on the market for severe asthma during the last decade. It prevents the linkage of the IgE and the receptors, thereby inhibiting mast cell degranulation. In clinical practice omalizumab significantly reduced the asthma exacerbations as well as the concomitant use of oral glucocorticoids.

In the “Th2-high asthma” phenotype, the hallmarks are increased levels of eosinophils and other markers (such as periostin). Because anti-IL-5 in this condition plays a crucial role in driving eosinophil inflammation, this cytokine or its receptors on the eosinophil surface has been studied as a potential target for therapy.

Two different anti-IL-5 humanized monoclonal antibodies, mepolizumab and reslizumab, have been proven effective in this phenotype of asthma (recently they both came on the market in the United States), as well as an anti-IL-5 receptor alpha (IL5Rα), benralizumab.

Other monoclonal antibodies, targeting different cytokines (IL-13, IL-4, IL-17 and TSLP) are still under evaluation, though the preliminary results are encouraging.

Finally, AIT, Allergen Immunotherapy, a prototype of Precision Medicine, is considered, also in light of the recent evidences of Sublingual Immunotherapy (SLIT) tablet efficacy and safety in mite allergic asthma patients.

Given the high costs of these therapies, however, there is an urgent need to identify biomarkers that can predict the clinical responders.

## Background

The evolution of asthma treatment was described by von Mutius & Drazen [1] and Bjermer [2] who pointed out how the new treatments were paralleled to the understanding of new pathogenetic mechanisms. This paper in a series by the WAO Collaborative on Severe Asthma (COSA) proposes a concise revision of how to approach and treat the eosinophilic pulmonary disorders in light of the new knowledge on the topic.

Undoubtedly, severe asthma is the most impacting “Pulmonary Eosinophilic Disorder” in terms of prevalence. Although severe asthma accounts for just 5 to 10% of total patients with asthma, it still represents a remarkable number of patients [3]. We should consider that not all severe asthmatic patients have eosinophilic inflammation; in fact, different patient phenotypes and/or endotypes are considered nowadays. This is the correct approach in selecting and diagnosing patients who are eligible for current and pending biologics for asthma treatment [4].

The careful and appropriate selection of patients is also the basis for successful allergen immunotherapy (AIT). Actually, asthma was not intended as a target disease for AIT, for a long time. In fact, asthma guidelines are not considering it either. The recent evidence of house dust mite tablets in both Europe [5, 6] and the United States [7] strongly supports the efficacy of sublingual AIT in asthma. The clear evidence will prompt the inclusion of AIT in asthma guidelines, always keeping in mind the concept of the evidence related to the single product(s) and not as a class effect, as reported recently in a statement of the World Allergy Organization on this specific issue [8].

How biologics or other treatment can target eosinophilic inflammation is the focus of this paper.

## Pharmacologic treatment of eosinophilic disorders

### Introduction

Asthma is a chronic inflammatory disorder of the airways characterized by variable air flow obstructions and tissue remodeling, mediated by a variety of inflammatory mediators and immune cells, including mast cells, several T cell subpopulations, eosinophils, basophils and neutrophils [9]. There is now recognition that distinct subgroups of asthma termed *endotypes* exist. An endotype, is “a subtype of a condition defined by distinct pathophysiological mechanisms” [10]. The American Thoracic Society (ATS)/European Respiratory Society (ERS) Task Force on Severe Asthma has defined this condition as asthma requiring global initiative for asthma step 4 or 5 treatment (high-dose inhaled corticosteroids and long-acting β-agonists or leukotriene modifier or tiotropium). Treatment options for severe asthma are limited and include Omalizumab, indicated in a selected phenotype of patients with high serum IgE levels [11], oral glucocorticoids and, more recently, tiotropium [12, 13].

Current research in severe asthma therapy is focused on the development of treatments that target specific components of airway inflammations. “Th2-high” asthma is characterized by increased levels of Type 2 inflammation in the airways including eosinophilia, increased numbers of airway mast cells and overexpression of periostin [14]. “Th2-high asthma” is characteristic of responsive to inhaled corticosteroids (ICS), whereas “Th2-low” asthma (classified as having low levels of type 2 inflammation) is not [15].

Several groups have reported cluster analyses of patient cohorts to investigate disease endotypes [16–21]. However, these studies are often limited by a lack of robust statistical validation or have generated clusters the identities of which are dominated by predominantly clinical parameters. Recently, large severe asthma cohorts were analyzed by using real-word assays already accessible to clinicians. This study identified six clusters based on blood and induced sputum measures [22]. The identification of additional biomarkers will provide more insights in the definition/selection of phenotype(s) eligible to a single therapy [4].

### Pharmacologic treatment of severe asthma

Pharmacologic treatment of severe asthma is based on the association of one of different medium- or high- dose inhaled corticosteroids (ICS) (Budesonide, Fluticasone, Beclomethasone, Ciclesonide and others) and long-acting β-adrenergic bronchodilators (LABA) (Formoterol, Salmeterol, Vilanterol, Indacaterol, and others). This approach has shown efficacy in the management of severe asthma and is recommended by Global Initiative for Asthma (GINA) guidelines. Patients with severe asthma may also be receiving as-needed short-acting β agonists (SABA). Racial differences in the response to β-agonists have also been reported [23].

Some patients with severe asthma remain symptomatic despite maximal recommended treatment. Tiotropium, a long-acting inhaled anti-cholinergic agent, significantly improves lung function in severe asthma [24–26]. There is some evidence that long-acting muscarinic antagonists (LAMA) added to ICS show some benefits over LABA plus ICS on some measures of lung function [27].

The leukotrienes modifier Montelukast is not as effective as LABAs when added to ICS in preventing asthma exacerbation or improving symptoms [28]. Whether individuals with the phenotype of aspirin-sensitive asthma respond better to leukotriene inhibitors than those without aspirin sensitivity has not been addressed.

Roflumilast, a selective phosphodiesterase 4 (PDE_4_) inhibitor, provides some improvements in lung function in patients with moderate-to-severe asthma [29]. In this study Roflumilast was used in combination with Montelukast in patients with uncontrolled asthma despite a moderate dose of ICS and LABA. This pilot study deserves additional investigations.

Bronchial thermoplasty (BT) was proposed as a technique to reduce airway stiffness and excessive narrowing [30]. Although the mechanism of action has not been elucidated, some positive outcomes in asthma have been reported [31, 32]; recently, a positive perspective of cost/effectiveness of this treatment has been envisaged [33].

### Treatment of eosinophilic asthma

Increasing evidence suggests that airway neutrophilia and eosinophilia represent two distinct inflammatory networks that contribute separately to severe asthma symptoms [16]. Interestingly, more than one eosinophilic or neutrophilic clusters were identified. One cluster was characterized by high serum periostin and IgE levels. The most neutrophilic disease was characterized by strong correlation between sputum YKL-40 and IL-8 levels, in addition to several markers of neutrophilic inflammation. Like others [34], they found no evidence of dysregulation of airway IL-17 in any subgroup, implying this cytokine might not be a promising target [35].

Targeting IL-5 or IL-5Rα is an appealing approach to the treatment of severe eosinophilic asthma [36]. Two different humanized anti-IL-5 monoclonal antibodies (Mepolizumab and Reslizumab) have been shown safety and efficacy in clinic trials of severe asthma. Mepolizumab has a glucocorticoid-sparing effect, reduces exacerbations and improves FEV1 and ACQ-5 score [37, 38]. Mepolizumab has been approved by FDA and EMA as an add-on maintaining treatment for adults with severe eosinophilic asthma. More recently, FDA approved Reslizumab with the same indication.

Reslizumab, a humanized anti-IL-5 monoclonal antibody, reduces blood and sputum eosinophils and improves FEV1 and ACQ score in patients with severe asthma [39]. The FDA recommended approval for Reslizumab in 2015 as an add-on maintaining treatment for adults with severe eosinophilic asthma.

Benralizumab is a humanized monoclonal antibody anti-IL-5Rα which binds to IL-5Rα on human eosinophils and basophils. Benralizumab improved lung function asthma control and ACQ-6 score compared to placebo in severe eosinophilic asthmatics [40]. A single-dose of Benralizumab administered to patients with severe asthma resulting in emergency department reduced the exacerbations during the following 3 months [41].

Targeting GATA-3, an important transcription factor of the Th2 pathway, may be beneficial in a subgroup of severe asthmatic patients. A novel DNA enzyme that cleaves and inactivates GATA-3 messenger RNA (mRNA) has been shown to inhibit the late asthmatic response to allergens [42].

Asthma is a prominent clinical hallmark of eosinophilic granulomatosis with polyangiitis (EGPA), previously called Churg-Strauss syndrome. In a small group of steroid-dependent EGPA patients, anti-IL-5 mab, namely Mepolizumab, had a glucocorticoid sparing effect without improving the pulmonary function [43]. In a pilot study we have found that Omalizumab has a glucocorticoid-sparing effect while decreasing blood eosinophils and improving lung function in EGPA patients [44].

Other approaches with biologics are specifically reported in details in another section of this document.

We wish here to underline how in the recent years a substantial improvement in therapeutic options for treating eosinophilic disorders became available, as summarized in Table 1, which shows examples of targeted therapies in preclinical or clinical development in severe asthma.

**Table 1:** Examples of Targeted Therapies in Preclinical or Clinical Development in Severe Asthma

Strategy	Target	Drug	Biological or Clinical Effects	References
Cell surface protein	Siglec-8	Anti-Siglec 8 monoclonal antibody	Apoptosis of eosinophils	Nutku *et al.*, Blood 101: 5014, 2003 [107] Bochner *et al.*, Clin. Exp. Allergy 39: 317, 2009 [108]
	CD172a		Inhibitor of signaling	Verjan Garcia *et al.*, J. Immunol. 187: 2288, 2011 [109]
	CD300a		Activation of inhibitory receptor	Munitz *et al.*, Blood 107: 1996, 2006 [110]
	Immunoglobulin-like receptor B			Munitz *et al.*, Blood 111: 5694, 2008 [111]
	α_4_β_1_, α_4_β_7_	Natalizumab	Increase blood eosinophils and inhibits their tissue accumulation	Abbas *et al.*, Neurology 77: 1561, 2011 [112]
	α_4_β_7_ integrin	Vedolizumab	No effect	Soler *et al.*, JPET 330: 864, 2009 [113]
	α_4_β_7_ , α_E_β_7_	Etrolizumab	Unknown	
	CCR3	GW766944	Block chemokine-induced eosinophils in vitro; no effect in vivo	Neighbour *et al.*, Clin. Exp. Allergy 44: 508, 2014 [114]
	CXCR2	SCH527123	Reduce blood and sputum neutrophils	Nair *et al*., Clin. Exp. Allergy 42: 1097, 2012 [115]
	CD52	Alemtuzumab	Deplete eosinophils in vivo	Wechsler *et al.*, JACI 130: 563, 2012 [116]
	CD131	CSL311	Unknown	
	CRTH2	0C000459	Reduce tissue eosinophils	Pettipher *et al*., Allergy 69: 1223, 2014 [117]
		ACT-453859	CRTH2 blockade	Géhin *et al.*, J. Clin. Pharmacol. 55: 787, 2015 [118]
	EMR1	Afucosylated anti-EMR1 monoclonal antibody	Deplete primate eosinophils	Legrand *et al.*, JACI 133: 1439, 2014 [119]
	Interleukin-4Rα	Dupilumab	Reduce airway eosinophils	NCT01312961
	Interleukin-4Rα	AMG-317	Do not reduce airway eosinophils	Corren *et al.*, Am. J. Resp. Crit. Care Med. 181: 788, 2010 [120]
	H4 Receptor	UR-63325 JNJ 28610244		Salcedo et al., Front Biosci 5: 178, 2013 [121] Dib et al., JLB 96: 411, 2014 [122]
Soluble mediator antagonist	Eotaxin-1	Bertilimumab	Inhibit Eotaxin-1 mediated eosinophil activation in vitro	Ding *et al.*, Curr. Opin. Investig. Drug 5: 1213, 2004 [123]
	IgE	Omalizumab	Reduces eosinophils at sites of allergic inflammation and peripheral blood	Detoraki *et al.*, J. Asthma 53: 201, 2016 [44]
	IL-4	Altrakincept Pascolizumab Pitrakinra	Reduce eosinophils at sites of allergic inflammation	Borish *et al.*, Am. J. Resp. Crit. Care Med. 160: 1816, 1999 [124] Hart *et al.*, Clin. Exp. Immunol. 130: 93, 2002 [125]
	IL-13	Tralokinumab Lebrikizumab Anrukinzumab RPC4046 QAX576	Reduce eosinophils in blood and at sites of allergic inflammation	Blanchard *et al.*, Clin. Exp. Allergy 35: 1096, 2005 [126] Maselli *et al.*, J. Asthma Allergy 8: 87, 2015 [127]
	Interleukin-17RA	Brodalumab	No effect	Busse *et al*., Am J Respir Crit Care Med 188: 1294, 2013 [35]
	TSLP	AMG157	Reduce eosinophils in blood and at sites of allergic inflammation	Gauvreau *et al.*, NEJM 370: 2102, 2014 [57]
Transcription factor	GATA3	SB010	Reduce IL-5 and late asthmatic response after allergen challenge	Krug *et al.,* NEJM 372: 1987, 2015 [42]

## Monoclonal antibodies in severe asthma

### Introduction

Severe refractory asthma is present when asthma remains uncontrolled despite maximal treatment [45]. The release of pro-inflammatory proteins mediates the inflammatory response associated with severe asthma. The identification of these mediators has resulted in the strategy of targeting these molecular pathways with monoclonal antibodies (hMab) for the treatment of severe asthma.

### Anti-IgE monoclonal antibody

The initial hMab (Omalizumab) developed for the treatment of asthma was directed against immunoglobulin E (IgE). Omalizumab prevents cross-linkage of the high-affinity IgE receptor, FcεRI, thereby inhibiting the degranulation, pro-inflammatory mediator release and production of newly formed lipid mediators from mast cells. It is effective in reducing oral corticosteroid dependence and exacerbation rates in allergic asthmatics with an elevated IgE level [46]. Omalizumab treatment rarely causes anaphylaxis [47], and an initial concern about increases in malignancy with its use was refuted in a large safety study [48]. Ligelizumab is another anti-IgE hMab, currently under development, which has a higher affinity binding for IgE when compared with Omalizumab. Allergen-induced bronchoconstrictor responses and skin prick test responses were also more effectively suppressed by Ligelizumab compared with Omalizumab [49].

### Anti-IL-5 monoclonal antibodies

IL-5 is essential for the development, differentiation, recruitment, activation and survival of eosinophils. Two hMabs have been developed which bind IL-5 and prevent its engagement with its receptor. These are Mepolizumab and Reslizumab. Both have been shown to reduce severe asthma exacerbations, improve FEV_1_, and allow oral corticosteroid reduction in severe asthmatics with persisting airway eosinophilia [38, 39, 50, 51]. Another hMab, Benralizumab, binds to IL-5R alpha and causes (as it is afucosylated) enhanced antibody-dependent cell-mediated cytotoxicity of both basophils and eosinophils [40, 52]. Both Mepolizumab and Reslizumab are now approved as add on therapy in patients with severe eosinophilic asthma.

### Anti-IL-4/IL-13 monoclonal antibodies

IL-4 and IL-13 are type 2 cytokines that have a variety of effects, including immunoglobulin class switching for the production of IgE, enhanced airway smooth muscle contractility, eosinophil recruitment and airway mucus production. Two hMabs (Lebrikizumab and Tralokinumab), which directly bind IL-13, are in clinical development. Both have been demonstrated to improve lung function and possibly reduce asthma exacerbations, but only in patients with severe asthma with biomarker evidence of a type-2 phenotype, demonstrated by elevated serum periostin levels or elevated IL-13 levels in sputum [53, 54].

IL-4 and IL-13 share part of the heterodimeric receptor complex (IL-4Rα). Dupilumab is a mAb to IL-4Ra that inhibits both IL-4 and IL-13 signaling. In an early phase clinical trial, treatment with Dupilumab allowed reduction of maintenance treatment with inhaled corticosteroid (ICS) and long-acting inhaled beta-agonist (LABA) treatment without loss of asthma control in patients with elevated blood eosinophil levels [55].

### Anti-Thymic Stromal Lymphopoietin (TSLP) monoclonal antibodies

TSLP is an interleukin-7-related cytokine secreted by airway epithelial cells. TSLP activates dendritic cells to release chemokines that recruit and activate Th2 cells. TSLP is increased in the airway epithelium of patients with severe asthma [56]. AMG 157, an anti-TSLP hMab, attenuated allergen-induced early and late asthmatic responses in mild allergic asthma. It also reduced baseline blood and sputum eosinophil counts and fractional exhaled nitric oxide (FeNO) concentrations before allergen challenge [57]. This suggests that TSLP is constitutively released in the airways of allergic asthmatic, and as it is “upstream” of the events leading to the release of type-2 cytokines, IL-5 and IL-13, its blockade may provide similar clinical benefit to blockade of both of these cytokines.

### Anti-IL-17 monoclonal antibodies

Th17 cells are CD4 T cells that express IL-17A, −17E, −17 F, and −22, and are able to mediate neutrophil activation. Overproduction of these IL-17 cytokines has been demonstrated in patients with severe neutrophilic asthma disease [58, 59]. Brodalumab, is an anti-17 receptor hMab, which had no effect on asthma control scores, symptom-free days, and FEV1 in patients with inadequately controlled moderate-to-severe asthma, who were also receiving inhaled corticosteroid therapy [35].

### Anti-TNF αlpha monoclonal antibodies

Initial studies in severe asthma using hMabs which bind TNFα showed promise [60, 61]; however, an increase in infections and malignancies during treatment with one anti-TNF hMab, Golimumab, when compared with placebo has halted further studies in asthma of this class of anti-Th1 targeted therapies [62].

### Conclusions

Asthma is a heterogeneous disease and this is particularly true of severe refractory asthma. Biologic treatments have shown promise in several phenotypes of severe asthma. Anti-IgE hMabs are effective in severe allergic asthma and hMabs directed against IL-5 in severe eosinophilic asthma, particularly in reducing severe asthma exacerbations. Other approaches directed against the IL-4/IL-13 and TSLP are under investigation.

## Specific immunotherapy in asthma, an example of personalized medicine?

### Background and introduction

In the available guidelines and systematic reviews it is stated that allergen immunotherapy (AIT) is not specific for the disease (rhinitis/asthma) but only for the allergen causing the disease itself [63]. This is reasonably based on the knowledge of the pathophysiology of allergic diseases and of the mechanisms of action of AIT [64]. Nonetheless, the efficacy and safety aspects tend to be kept separate for asthma and rhinitis, as testified by several large reviews and meta-analyses [65–72]. Allergic asthma, in particular, remains one of the most important matters of debate for the use of AIT [73, 74]. From a historical point of view, the scepticism towards AIT in asthma is mainly due to the reports of severe (or fatal) reactions in asthmatic patients, when only the subcutaneous route (SCIT) was available [75–78]. The perspective partially changed after the sublingual route (SLIT) of administration became available, largely used and studied. In fact, with SLIT a satisfactory efficacy could be achieved also in asthmatic patients, and no severe adverse event or fatality was reported [79, 80]. In addition, it should be taken into account that most of the severe events (and rare deaths in asthmatic patients), were described in the U.S. surveys, where high concentrations of allergens and mixtures of allergens were used [81].

It is also true that none of the trials evaluating asthma symptoms was adequately designed and reported [73, 74]: none of the trials had a sample size calculation and a power analysis based on asthma symptoms or pulmonary functions as primary outcome. Moreover, there is no formal consensus on which measurement parameters for asthma should be chosen. Asthma symptoms, rescue medications, combined scores, asthma-free days, forced expiratory volume in one second (FEV1), and asthma exacerbations are all equally reasonable choices. In this regard, objective functional pulmonary measurements were carried out only sporadically. More recently, some clinical trials were specifically designed for asthma, and took into account specific asthma-related parameters (such as the use of inhaled corticosteroids or the exacerbation rate). Finally, we should consider that isolated allergic asthma without rhinitis is rare, whereas asthma is present in more than 30% of patients with rhinitis [82]. In fact, the majority of recent clinical trials were performed in allergic rhinitis (with or without allergic asthma), and very few trials were specifically designed to evaluate the effect of AIT in asthma alone. Nowadays, the principal questions are: is AIT effective in asthma or does AIT provide an adjunct benefit in asthma?; does AIT worsen asthma?; is asthma an absolute contraindication and a risk factor for adverse events during AIT?

### SCIT and SLIT in asthma: overview of clinical trials

#### Clinical efficacy

It is not possible to describe in detail herein each of the studies of AIT in asthma, and summaries of data in literature are already available [65–74]. Thus we are quoting only the meta-analyses which specifically dealt with asthma, as a comprehensive example of the results achieved so far (with their limitations).

There are numerous clinical trials of SCIT which considered asthma, most of them published before 1990 and generally including small numbers of patients. The available largest meta-analysis [69] included 88 randomized controlled trials (70 of which placebo-controlled) published between 1954 and 2009. The methodological quality, was low to moderate, and very few trials achieved the maximum score. Symptom scores were reported in 35 studies, medication scores in 21 and 20 studies had a pulmonary function measurement. This meta-analysis reported a borderline reduction in symptoms with mite allergens, but an apparent effect with pollens. The effect on asthma medication intake was overall significant. No change in pulmonary function could be seen, whereas a decrease in allergen-specific bronchial response was consistently shown.

The number of randomized controlled trials for SLIT is quite large and, therefore, some meta-analyses were performed. The first meta-analysis specifically focusing the effect of SLIT in asthma was published in 2006 [65], and analysed 25 trials with 1076 patients (adults and children). This meta-analysis reported a significant difference between SLIT and placebo for categorical outcomes (better / unchanged / worsened), but no difference using the symptom or medication scores for asthma. On the other hand, the combined symptom score of asthma-rhinitis were clearly in favour of SLIT. Nonetheless, there was a high heterogeneity, which limits at some extent the positive conclusions. Another meta-analysis [67] dealt with paediatric asthma (patients < 18 years) and included nine trials with 441 patients. The results, according to the mean standardized difference demonstrated a significant reduction in both symptoms (P = 0.02) and medication use (P = 0.007) vs. placebo. Also in this case a high degree of heterogeneity was found, due to the variable inclusion criteria, scoring systems and regimens. Two other meta-analyses, one restricted to mite extracts [70] and one to grass extracts [71] were also published The meta-analysis for dust mite included nine trials with asthma symptoms/medications and the results showed a significant reduction vs placebo in both symptom scores (P = 0.02) and medication intake (P = 0.02). The meta-analysis for grasses did not report specific results for asthma. Of note, for SCIT only one “large trial” was performed [83].

Only few recent studies [5, 84] were specifically designed to assess asthma related outcomes. Zielen et al. [84] evaluated 65 asthmatic children (GINA step 2–3), randomized to inhaled fluticasone alone or plus mite-SCIT. After 2 years of treatment, the dose of inhaled fluticasone to maintain asthma controlled was halved in the active versus the control group. In another study [5] performed in >600 mite-allergic adolescents and adults, with a dose-ranging design, a significant reduction in the dose of inhaled corticosteroids could be demonstrated with the highest dose of SLIT. Wang et al. [85], in 484 adult subjects receiving mite-SLIT in a randomized controlled trial, found a significant difference in asthma control only in moderate asthma, but not in mild disease. This latter finding is in line with previous observations: if asthma is per se well controlled by drugs, or patients are almost symptom-free, no or marginal effects of AIT can be appreciated [86].

#### Safety

In the studies published so far available, the frequency of SCIT-induced systemic reactions (SRs) is largely variable according to the allergen, the administration schedule, standardization of the extract, the maintenance dose given and the severity and type of disease. Another main problem is the lack of a universally accepted classification/grading of the adverse events. This problem has recently been addressed by the World Allergy Organization with the proposal of new classification and grading systems [87, 88]. Finally, most data on systemic side effects come from small controlled studies, whereas only large-scale surveys can assure reliable data on the prevalence, characteristic and severity of side effects. The majority of the data we have available on the safety of SCIT derive from the large surveys performed in the United States [76–78, 89]. Overall, these surveys recorded about 50 deaths over a 50-year period with a risk of one death every 2.500.000 injections and one near-fatal reaction per million injections. Again, it is important to remember that the clinical practice of SCIT in the United States is different from Europe, as mixtures of multiple allergens are commonly used [81]. Thus, caution should be applied in transferring the USA data to other countries. Less data are available for Europe. After the well-known report of 26 deaths in UK in 1986 [75], probably ascribable to incorrect practice in most cases, fatalities have become extremely rare, and no report was released in the last decade. Several surveys [90–93], reported an overall rate of systemic reactions of about 5% of patients. The more recent multicentre survey [93] reported that systemic reactions were slightly more frequent in rhinitis with asthma than in rhinitis alone.

Overall the safety of SLIT is superior to that of SCIT [79, 80]. This is testified to by the reports of “large trials” including hundreds of patients, and no fatality has been so far reported with SLIT in more than 30 years of clinical trials and practical use. Interestingly, a study by Dahl et al. [94] specifically assessed the safety of SLIT in asthma. More than 100 grass-allergic asthmatics were enrolled and studied before the pollen season. The number of side effects linked to asthma (wheezing cough, dry throat, dyspnoea, increased bronchial secretion) was similar between the active and placebo group, and there was no evidence of asthma aggravation. In general, SLIT induced asthma only rarely. In another study, the progressive increase of doses increased local AEs, but not asthma symptoms [95].

Despite the overall rarity of deaths the surveys agreed on the fact that uncontrolled asthma is the most prominent risk factor for fatalities and severe adverse events, including asthma itself. Thus, asthma, if well controlled it is not an absolute contraindication to AIT [96].

#### The additional effects of AIT in asthma

There is another important aspect to be considered when evaluating AIT in asthma, that is the preventive effect. Rhinitis is the major risk factor for the development of asthma [90, 97] and AIT, as biological response modifier can interfere with the progression from rhinitis to asthma. Indeed, the preventative effect of AIT (reduction in the risk of developing asthma) was suggested about 50 years ago in an observational open prospective study [98], but it was confirmed in more rigorous trial only in the last decades [99–101]. The Preventative Allergy Treatment study enrolled 205 children (aged 6–10 years) suffering from allergic rhinitis. They were randomized to either drug therapy alone or drugs plus SCIT. After 3 years, the SCIT-treated patients had developed significantly less asthma than the control group, with an odds ratio of 2.5. Interestingly, the beneficial effect of SCIT lasted several years after discontinuation, and also at the 10-year follow-up there were significantly less patients with asthma in the formerly SCIT-treated group [99]. Concerning SLIT there are two studies supporting the possible disease-modifying effect of the treatment. The first open controlled study [100] involved 113 children aged 5–14 years suffering from seasonal rhinitis due to grass pollen. They were randomly allocated to medications plus SLIT or medications only. After 3 years, 8/45 SLIT subjects and 18/44 controls had developed asthma, with a relative risk of 3.8 for untreated patients. Another randomized open controlled trial [101] involved 216 children (age 5–17 years) suffering from rhinitis with/without intermittent asthma. They were randomly allocated 2:1 to drugs plus SLIT or drugs only, and followed for 3 years for the presence of persistent asthma. The prevalence of persistent asthma was 2/130 (1.5%) in the SLIT group and 19/66 (30%) in the control group, with a number to treat of 4. These data support the need for further randomized blinded trials of the potential long term benefits of SLIT in the development of asthma [102].

#### AIT as “personalized” or “precision” medicine

**Fig. 1:**
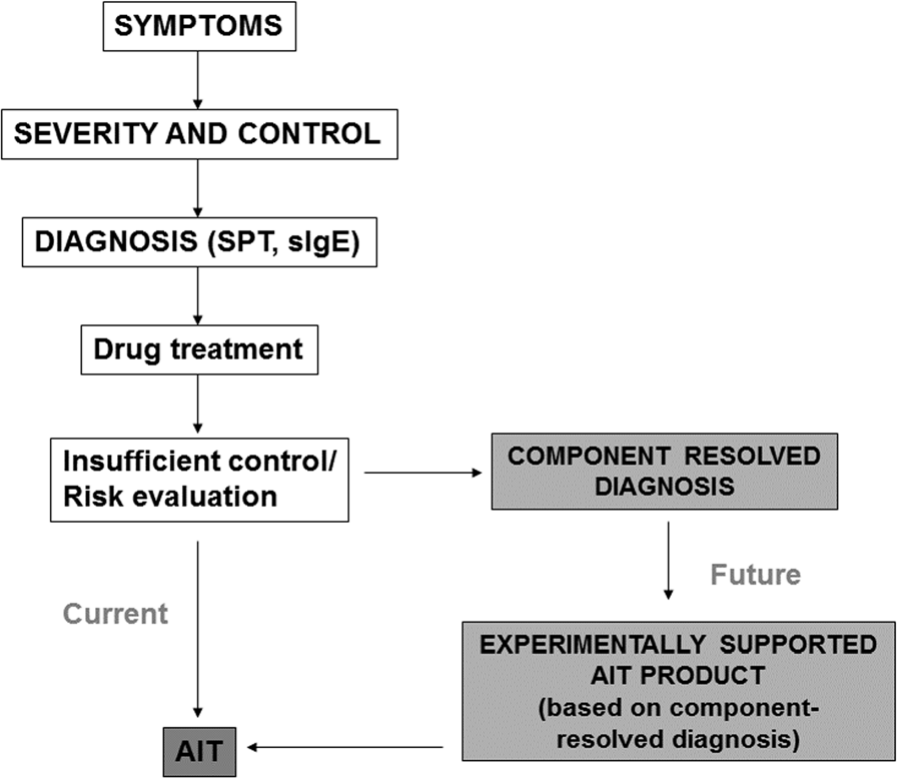
Current and possible future approach in prescribing AIT for respiratory allergy (SPT = skin prick test: sIgE = specific IgE) (Modified from Canonica GW et al., World Allergy Organ J 2015) [103]

**Fig. 2:**
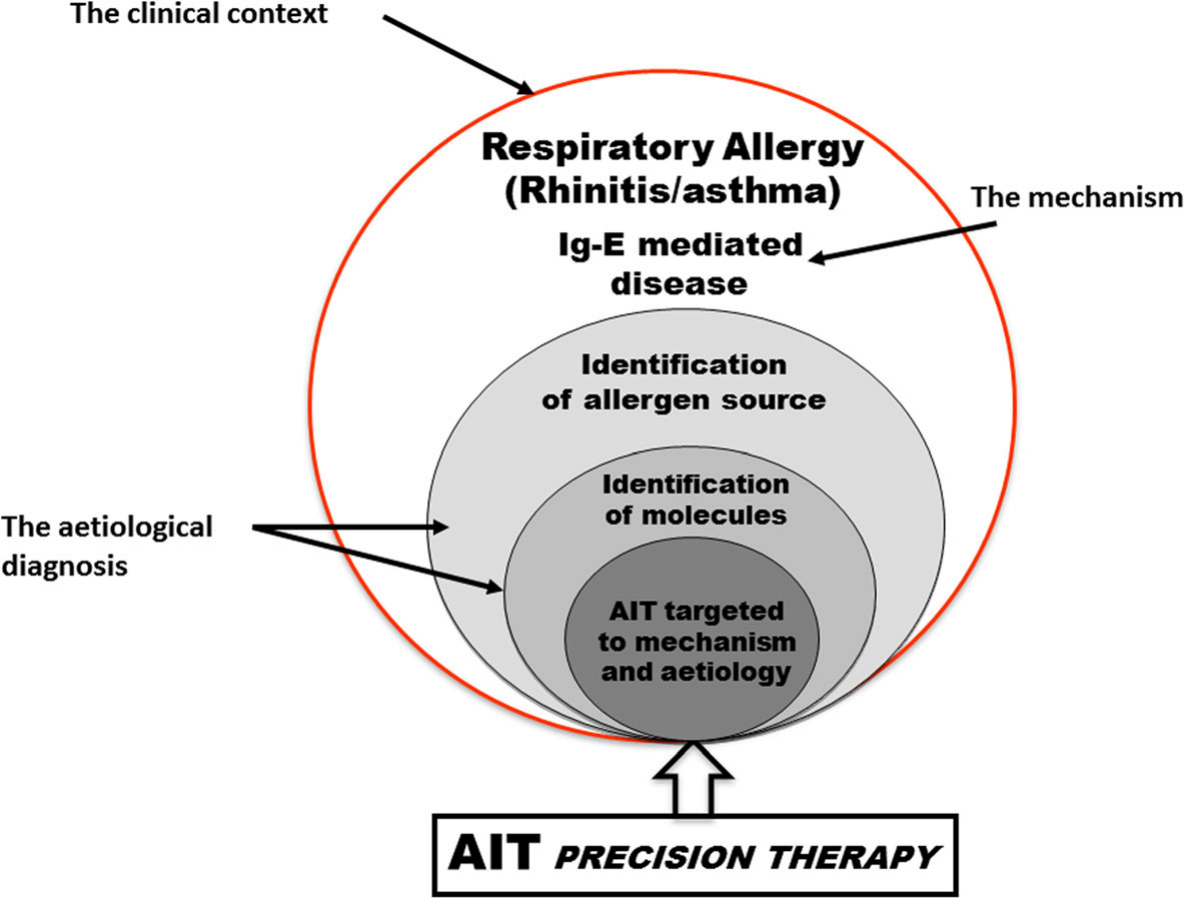
AIT as a model of “precision medicine” (Modified from Passalacqua G et al., Clin Molecular Allergy 2015) [104]

The current view in medicine is that of a “personalized” or “precision” approach. The “blockbuster approach” (i.e. one size fits all) cannot be currently used with many of the very expensive treatments available, where the best cost/effective treatment should be provided. The “precision medicine” can be defined as a structural model aimed at customizing healthcare at best, with medical decisions, practices, and/or products tailored on an individual patient (Fig. 1) [103]. In other terms, the underlying mechanisms of a given disease should be clearly defined. Then the biomarkers for that disease should be identified and, subsequently, the targeted therapeutic approach can be chosen. This implies that the response to a given therapy can be predicted a priori, by means of specific biomarkers. AIT represents a good paradigm of this approach. In such case, we have: a) the clinical aspects well defined and easy to diagnose; b) the mechanisms (IgE-mediated) are well identified:; c) reliable diagnostic tests are available, including the molecular aspects [103]; d) AIT is allergen-oriented [104] (Fig. 2). Nonetheless, we still need biomarkers predictive of the expected efficacy and, consequently, the identification of the eligible patients, with direct economic implications. Certainly we would need a more spread knowledge on molecular allergy, to be ourselves more adherent to the definition of personalized medicine. Moreover, a clear characterization and definition of commercial products for vaccination is also urgently needed. A precision medicine requires precision approaches, whereas nowadays, for many commercial products the characterization remains poor, and in some cases an experimental proof of efficacy is lacking [8].

### Conclusions

In recent years, evidence-based medicine has become more and more important, and almost all guidelines and recommendations are prepared according to well accepted evaluation systems such as the GRADE [105]. The evidence based criteria firstly require that the methodology of trials is adequate, and this is not always the case for AIT studies. Almost all the available randomized controlled trials suffer from methodological limitations: small number of patients, absence of a sample size calculation based on a definite outcome, large variability in methodology. This even better applies to asthma studies, where only few trials were adequately powered [106]. Based on the earliest clinical trials xand surveys, only uncontrolled asthma remains the main contraindication to AIT, whereas in controlled allergic asthma it can be confidently used, together with medications, when the causal role of the allergen (pollens, mite or pets) is clearly confirmed. The recommendation of not giving SIT in patients with severe uncontrolled asthma remains valid for both SCIT and SLIT. When asthma is the only manifestation of allergic disease, AIT may exert a beneficial effect, at least by reducing the need for medications and reducing associated bronchial responsiveness. This does not imply that AIT is to be used as the primary therapeutic approach in adults or indeed in children, where highly effective drugs (i.e. inhaled steroids) are currently available. Future studies are needed to focus patients with moderate/severe asthma, where asthma is the primary outcome. Long-term benefits after discontinuation need to be evaluated as well.

## Conclusions

Although the prevalence of severe asthma is relatively low, it accounts for 50% of the global costs of the disease, and it is responsible for the majority of the hospitalizations and Emergency Department accesses. For these reasons research has been focused on this condition.

According to the GINA Guidelines the pharmacologic treatment is currently based on the combination of a high dose of ICS with LABA. In non-responsive patients the addition of anti-cholinergic drugs such as tiotropium can be an effective option.

However the recent identification of different endotypes of asthma, based on the inflammatory pattern has led to a different approach to the treatment, which targets the specific components of the inflammation. Omalizumab prevents the cross-linkage of the IgE and the high-affinity IgE receptors (FcεRI), thereby inhibiting mast-cell degranulation. Its efficacy in reducing exacerbations as well as the oral use of CS has been demonstrated in severe allergic asthma.

Besides this allergic phenotype the “Th2-high asthma” is characterized by increased levels of Type 2 inflammation in the airways including eosinophilia and overexpression of periostin. In this condition IL-5 has a pivotal role in driving eosinophilic inflammation and therefore targeting IL-5 or Il-5R alpha is an attractive biologic approach.

Two different humanized anti-IL-5 monoclonal antibodies (mepolizumab and reslizumab) have been proven effective and safe in severe asthma, in patients with more severe eosinophilic inflammation. In addition, benlarizumab, a humanized antibody anti IL-5R alpha, which binds the receptors on human eosinophils and basophils, has also significantly reduced the exacerbations in patients with eosinophilic severe asthma over a three-month treatment period.

Several monoclonal antibodies targeting other proinflammatory interleukins (IL-4 and IL-13) are still under evaluations.

The anti-IL-17 receptor monoclonal antibody, brodalumab, is currently the only biologic treatment under evaluation in neutrophilic asthma.

With the potential use of these biologic drugs a tailored approach in severe asthma will be possible in the future. However, given the high costs of these treatments, the economical sustainability of this approach needs a parallel investigation of clinical and/or biological marker of efficacy of these drugs in order to use in every case the right treatment in the right patient.

## Key points

- The prevalence of severe asthma is low (5-10%), but it accounts for most of ER admissions and hospitalizations due to the disease. Moreover, this condition is responsible for 50% of the costs of the disease.
- The knowledge of the biological mechanisms of the inflammation underlying the disease has led to a different approach of the treatment, based on the use of monoclonal antibodies which can interfere with the proinflammatory cytokines.
- Omalizumab, an anti-IgE monoclonal antibody, significantly reduces exacerbations and the use of oral glucocorticoids.
- In Th2-high asthma eosinophil and IL-5 have a pivotal role. Encouraging results have been reported in studies where patients with severe eosinophilic asthma were treated with anti-IL-5 (mepolizumab and reslizumab) or an anti IL-5Rα (benralizumab) antibodies.
- Anti-IgE monoclonal antibody (omalizumab) is established as a treatment option for severe allergic asthma.
- Anti-IL-5 monoclonal antibodies (mepolizumab; reslizumab) are approved for the treatment of severe eosinophilic asthma.
- Anti-IL-13, anti-IL4Rα and anti-TSLP monoclonal antibodies are currently being studied for severe eosinophilic asthma.
- Other biologics targeting different mechanisms are currently under investigation and will soon be available.
- Given the high costs of these therapies, there is a need to identify clinical and/or biological markers which can select positive responders to these treatments

## Abbreviations

AIT: Allergen immunotherapy
ATS: American Thoracic Society
BT: Bronchial thermoplasty
COSA: Collaborative on Severe Asthma
EGPA: Eosinophilic granulomatosis with poliangiitis
ER: Emergency Department
ERS: European Respiratory Society
FeNO: Fractional exhaled nitric oxide
GINA: Global Initiative for Asthma
hMab: Monoclonal antibodies
ICS: Inhaled corticosteroids
LABA: Long acting beta-adrenergic bronchodilators
LAMA: Long acting muscarinic antagonists
PDE4: Phosphodiesterase 4
SABA: Short acting beta agonist
SCIT: Subcutaneous immunotherapy
SLIT: Sublingual immunotherapy
SRs: Systemic reactions
